# Significant simultaneous changes in serum CA19-9 and CA125 due to prolonged torsion of mature cystic teratoma of the ovary

**DOI:** 10.1186/1477-7819-12-353

**Published:** 2014-11-22

**Authors:** Dong Soo Suh, Soo Hyun Moon, Seung Cheol Kim, Jong Kil Joo, Won Young Park, Ki Hyung Kim

**Affiliations:** Department of Obstetrics and Gynecology, Pusan National University School of Medicine, 179, Gudeok-Ro, Seo-Gu, Busan, 602-739 Korea; Biomedical Research Institute and Pusan Cancer Center, Pusan National University Hospital, 179, Gudeok-Ro, Seo-Gu, Busan, 602-739 Korea; Department of Pathology, Pusan National University School of Medicine, 179, Gudeok-Ro, Seo-Gu, Busan, 602-739 Korea

**Keywords:** CA19–9, CA125, Teratoma, Torsion

## Abstract

Mature cystic teratoma is a common benign neoplasm of the ovary. Complications occur in approximately 20% of cases. Clinical manifestations, laboratory findings, and imaging studies can assist in making a diagnosis of ovarian torsion of mature cystic teratoma. Furthermore, serum tumor markers may be helpful for diagnosing mature cystic teratoma and its torsion and, thus, can lead to early surgical intervention. A 56-year-old woman presented with a huge pelvic mass and pelvic pain. Serum CA19-9, CA125, and carcinoembryonic antigen levels were abnormally elevated at >700 U/ml, 282.5 U/ml, and 3.94 U/ml, respectively. The tumor was surrounded by extensive adhesions and showed inflammatory changes. The serum levels of these markers returned to normal levels after surgery.

## Background

Mature cystic teratoma (MCT) is the most common germ cell tumor of the ovary in women of reproductive age. Clinical characteristics of MCT have been well documented, and about 20% of women with MCT experience complications, such as torsion, rupture, infection, and malignant transformation. Ovarian torsion is a common complication and constitutes a surgical emergency, and the rate of torsion has been reported to range from 12.9% to 15% in MCT patients [1, 2]. Furthermore, ovarian torsion accounts for 3% of cases of acute abdominal pain that present at emergency departments [3]. Delay in recognition and treatment of torsion can have serious consequences such as peritonitis and even death [4]. Laboratory and imaging findings can assist the diagnosis of MCT and its complications, and serum tumor markers can provide additional information about the clinical features and complications of MCT when the differential diagnosis of an ovarian mass by imaging is limited.

Here we report a case of ovarian torsion of MCT with rapid, significant changes in the serum levels of CA19-9 and CA125 in a postmenopausal woman.

## Case presentation

**Figure 1:**
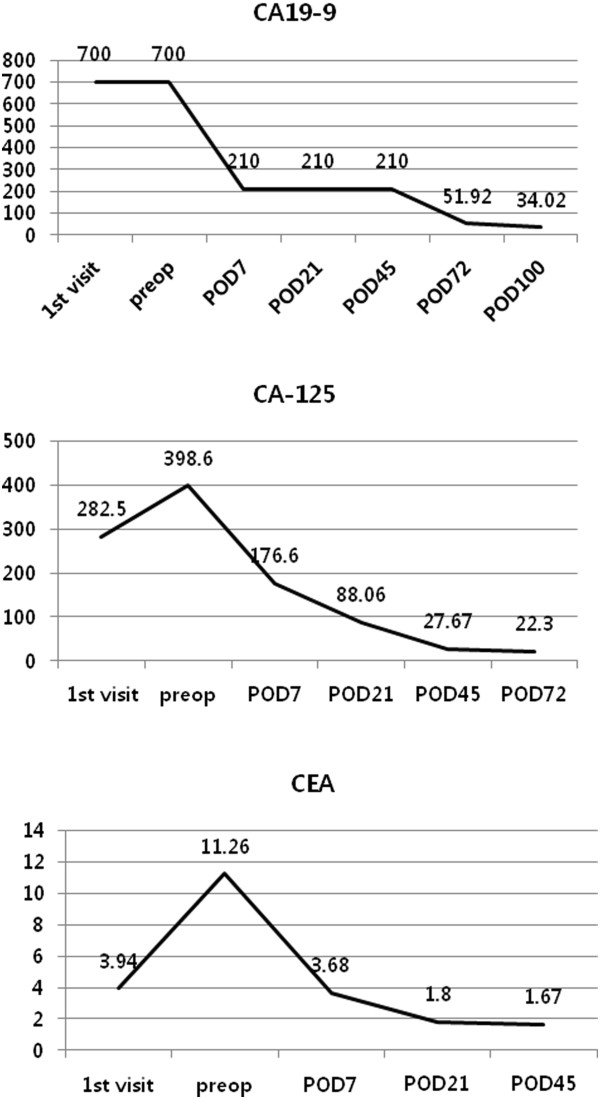
**Perioperative changes in serum tumor makers.** CA19-9, CA125, and carcinoembryonic antigen (CEA) levels (U/ml) were highly elevated preoperatively, but returned to normal levels after surgery. POD, postoperative day; preop, preoperative.

**Figure 2:**
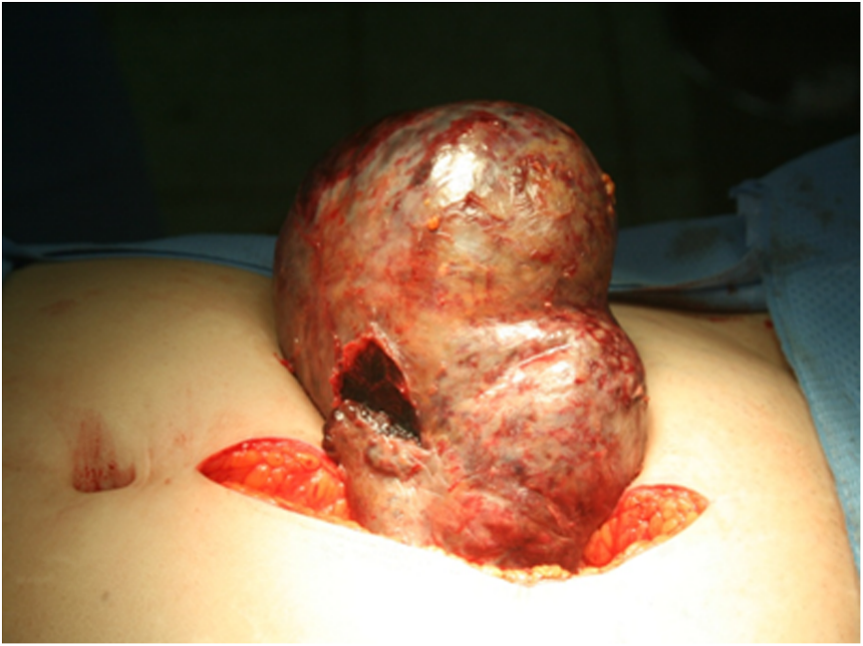
**Operative finding of right ovarian tumor showing a friable, discolored, and irregularly contoured surface.**

A 56-year-old postmenopausal woman presented with a huge pelvic mass. The patient had experienced moderate pelvic pain for 5 days before visiting a local clinic. Pelvic ultrasonography revealed a huge mass 11 cm in diameter in the right adnexa. Contrast-enhanced abdominopelvic computed tomography (CT) revealed an 11.0 × 7.5 cm mass containing a fat component arising from the right adnexa. She was referred to our hospital department due to abnormally high tumor markers and suspicion of a coexistent malignancy due to elevated serum CA19-9, CA125, and carcinoembryonic antigen (CEA) levels of >700 U/ml, 282.5 U/ml, and 3.94 U/ml, respectively. These serum markers were measured again preoperatively in our hospital and were found to be higher (Figure 1). Laboratory examination revealed increased white blood cell count (18,500/uL) and erythrocyte sedimentation rate (90 mm/hour). Previous CT findings were reviewed and showed asymmetric wall thickening of the mass and increased fat strands in peritoneum. During laparotomy, a huge right ovarian tumor that appeared dark brown presumably due to torsion was observed and was surrounded by extensive adhesions to omentum, rectum, and the posterior wall of the uterus. The surface of the tumor was friable, discolored, and had an irregular contour, suggesting inflammatory change, probably due to torsion-induced necrosis (Figure 2). Right salpingo-oophorectomy was performed and adjacent adherent omentum was excised. Frozen section of the tumor revealed MCT. Pathologically, most of the tumor was necrotic and compatible with MCT with hemorrhagic infarction and chronic inflammation of omentum. Following surgery the patient recovered without any complications, and serum levels of CA19-9, CA125, and CEA decreased to normal levels (Figure 1).

### Discussion

MCT is the most common benign neoplasm of the ovary. Adnexal torsion is a gynecologic surgical emergency, as delay in surgical treatment may lead to serious inflammatory consequences [4]. Sometimes the diagnosis of torsion is not straightforward because symptoms are nonspecific. Acute pelvic pain in torsion may be constant or intermittent depending on the degree of torsion. The episodes of pain can occur for several days to months prior to definitive treatment. In cases of partial torsion, there may be a history of similar transient episodes of pain. Furthermore, the pain can vary in intensity and may not be severe. If torsion is prolonged and treatment delayed, the adnexa may become necrotic and infected, and the patient may present with signs of peritonitis [5]. Some authors have concluded that the time between pain onset and definitive treatment is a marker of necrosis. Chen and colleagues showed that extensive hemorrhagic necrotic cases had torsion durations of 48 hours or longer [6], whereas Emonts and colleagues reported a median time from onset of pain until surgery of 5.3 days in patients with microscopic necrosis [7]. In our case, the patient first visited hospital 5 days after the onset of pain because the pain was tolerable, and surgery was performed 12 days after the onset of pain. The mass was found to be surrounded by extensive adhesions and the tumor surface exhibited inflammatory changes. The abnormally high serum tumor markers in our patient were probably due to prolonged torsion and subsequent peritoneal inflammation.

Ultrasonography is usually used for the diagnosis of ovarian MCT. However, accurate diagnosis is difficult because tumor contents vary. In addition, 80% of cystic teratomas have sonographic appearances similar to those of ovarian malignancies [8]. Therefore, the diagnosis of ovarian MCT requires additional diagnostic aids, such as tumor markers. Furthermore, some of tumor markers may be helpful in the diagnosis of torsion and, thus, lead to prompt treatment.

The rates of elevation of CA19-9 and CA125 have been reported to be 39.6 to 59% and 13.5 to 25%, respectively [1, 9, 10]. Emin and colleagues showed that CA19-9 is more frequently elevated than CA125, and hence a more useful marker of MCT [10]. On the other hand, CA19-9 is a widely used tumor marker for various malignancies, and is usually increased in gastrointestinal tumors. CA19-9 levels may have clinical significance in MCT of the ovary, and are not significantly related to age, body mass index, bilaterality, or serum CA125 levels [1]. Furthermore, elevated CA19-9 levels may be important for the diagnosis of MCT and appear to be correlated with tumor diameter [1, 11, 12], and the extent of tissue necrosis due to ovarian torsion [1, 2]. Kyung and colleagues demonstrated that serum CA19-9 levels in women with torsion were significantly higher than in those without torsion [1]. Previous studies have demonstrated that malignant transformation is not related to serum CA19-9 elevation, and that elevated CA19-9 levels in women with MCT of the ovary are not associated with malignant transformation [13].

CA125 is an extensively studied marker in the context of ovarian tumors. Serum CA125 levels provide information preoperatively that aid discrimination of benign and malignant adnexal masses. However, CA125 is not a tumor-specific antigen, and is also elevated in patients with endometriosis, adenomyosis, leiomyoma, and pelvic inflammatory disease. Accordingly, knowledge of CA125 alone is unhelpful for the diagnosis of MCT, and elevated CA125 levels in MCT may be related to peritoneal inflammation, such as that associated with pelvic inflammatory disease. In our case, the CA125 elevation was probably caused by torsion and inflammatory processes.

CA19-9, in combination with CA125, might be a useful marker for the discrimination of MCT from ovarian cancer [12]. In a retrospective study of 239 patients with ovarian MCT, it was found that elevation of CA19-9 alone was more frequent in patients with MCT than ovarian cancer, and that the simultaneous elevation of CA19-9 and CA125 was associated with a higher probability of a malignant neoplasm [12]. In another study, Fujiwara and colleagues observed that CA125 and CA19-9 levels increased dramatically prior to surgery following torsion of an ovarian tumor [14]. Our case showed simultaneous elevation of CA19-9 and CA125 that was not associated with ovarian malignancy; rather, they appeared to be associated with torsion of the ovary and resulting peritoneal inflammation.

The rate of elevation of CEA in ovarian MCT has been reported to range from 9.1 to 16.3% [9–11]. However, it is of little significance in MCT [9, 11].

Nevertheless, these three serum markers can provide valuable information for the diagnosis of MCT and for predicting its complication, although CA125 or CA19-9 testing is not sufficient for the diagnosis of MCT [10]. Our case suggests that concurrent abnormal elevations of CA19-9 and CA125 are manifestations of torsion of the ovary and subsequent necrosis and inflammation, which is consistent with the observations made in previous studies [2, 14]. The relationship between tumor markers and inflammation/ischemia has not been well documented. Only a few reports suggested that CA19-9 might have been stimulated by the inflammatory change due to the ischemic reaction after torsion and might predict the extent of tissue necrosis [2, 14]. The role of ischemia and necrosis was not fully defined. Further studies are needed to confirm the biochemical mechanisms of elevation of these markers.

In our case, prolonged torsion and delayed treatment were caused by a number of factors. First, symptoms were nonspecific, which delayed hospital visit. Second, the first gynecologist approached at the primary clinic under-interpreted CT findings and over-interpreted serum tumor marker findings. In particular, CT findings suggesting adnexal torsion and high tumor markers were interpreted as indicators of ovarian malignancy. Third, delayed referral to a tertiary center was attributed to adherence to a routine outpatient schedule.

## Conclusion

In conclusion, a highly elevated serum CA19-9 level may be an adjunct serum marker for the diagnosis of ovarian teratoma, and could also provide useful information on the presence of torsion. Furthermore, simultaneous elevations of CA19-9 and CA125 are not always associated with malignancy and may improve the detection of torsion and the prediction of the extent of necrosis. Perioperative assessment of tumor markers may provide valuable information on the progression or resolution of inflammatory processes following torsion of the ovary. The interpretation of tumor marker elevations should be made in light of the clinical condition. The dramatic changes in serum markers observed perioperatively in our patient gave us a better understanding of the inflammatory process following prolonged ovarian torsion.

## Consent

Written informed consent was obtained from the patient for the publication of this report and any accompanying images.

## Abbreviations

CEA: carcinoembryonic antigen
CT: computed tomography
MCT: mature cystic teratoma.
